# Physiotherapists and Osteopaths’ Attitudes: Training in Management of Temporomandibular Disorders

**DOI:** 10.3390/dj10110210

**Published:** 2022-11-04

**Authors:** Stefano Saran, Sabina Saccomanno, Maria Teresa Petricca, Andrea Carganico, Salvatore Bocchieri, Rodolfo Francesco Mastrapasqua, Elena Caramaschi, Luca Levrini

**Affiliations:** 1 Department of Human Sciences, Innovation and Territory, School of Dentistry, Postgraduate of Orthodontics, University of Insubria, 21100 Varese, Italy; 2 Department of Health, Life and Environmental Science, University of L’Aquila, Piazza Salvatore Tommasi, 67100 L’Aquila, Italy; 3 Department of Medical, Oral and Biotechnological Sciences, “G. d’Annunzio” University of Chieti–Pescara, 66100 Chieti, Italy; 4 ENT Department, Rivoli Hospital, ASL TORINO 3, 10098 Torino, Italy; 5 Private Practice, 43125 Parma, Italy

**Keywords:** temporomandibular disorders, physiotherapy, osteopathy, dentistry, awareness, knowledge

## Abstract

Temporomandibular disorders (TMDs) are a condition which has multifactorial etiology. The most acknowledged method to classify TMDs is the diagnostic criteria (DC) introduced firstly by Dworkin. This protocol considers different aspects that are not only biological, but even psychosocial. Diagnosis is often based on anamnesis, physical examination and instrumental diagnosis. TMDs are classified as intra-articular and/or extra-articular disorders. Common signs and symptoms include jaw pain and dysfunction, earache, headache, facial pain, limitation to opening the mouth, ear pain and temporomandibular joint (TMJ) noises. This study regards two kind of clinicians that started in the last years to be more involved in the treatment of TMDs: osteopaths (OOs) and physiotherapists (PTs). The purpose is to analyze their attitude and clinical approach on patients affected by TMDs. Four hundred therapists answered an anonymous questionnaire regarding TMJ and TMDs. OOs showed greater knowledges on TMDs and TMJ and, the therapists with both qualifications seemed to be most confident in treating patients with TMDs. In conclusion this study highlights OOs and all the clinicians with this qualification, have a higher confidence in treating patients with TMD than the others. Dentists and orthodontists, according to this study, should co-work with OOs and PTs, because they are the specialists more requested by them than other kinds of specialists.

## 1. Introduction

The temporomandibular joint (TMJ) is a synovial joint that allows the mandibular function which includes mastication, phonation and deglutition [1]. It presents a close relationship with many other biological structures. It is tied to the masticatory system muscles, lymphatic and nervous system [2]. The main facial muscles involved in the function of the TMJ are the masseter muscles, the pterygoids and the temporal ones [2].

TMDs are a muscle skeletal disorders affecting the TMJ, the masticatory muscles and associated structures including dental occlusion and cervical spine [3,4]. The American Academy of Orofacial Pain defines TMDs a collective term for several clinical problems [5], which can produce orofacial pain and functional disturbances [6]. TMDs signs and symptoms occur when the structural tolerance of the stomatognathic system is overreached [7]. They may include local pain in the TMJ and/or masticatory muscles, limited mouth movements, TMJ sounds and headache [8,9,10]. TMJ noises and sounds can be classified in clicks. The click’s timing is very important to understand the prognosis of the health issue. A precocious click while opening indicates a high level of dislocation of the disk from the condyle, instead a late noise means a less important dislocation. The opposite concept regards the closing act of the mouth [11]. It is known that TMJ noises do not mean surely a need of treatment, because in many cases there are minimum entities of dislocations not disturbing the patients in a concrete way [12]. Sometimes TMDs can seriously invalidate the patient’s quality of life and reduce the work ability [12,13]. This happen when symptoms and signs are continuous, but it is right to affirm that when TMDs are invalidating, possibly there are comorbidities that exacerbate pain and reduce pain tolerance [13]. The most common comorbidities are disease like fibromyalgia: a systemic condition with widespread muscle and joint pain in different areas of the human body. Fibromyalgia also favors depression and other behavioral disfunctions, that even characterize the subjects with TMDs [13,14]. Other health problems that may be present in TMDs’ patients are gut problems, like irritable bowel syndrome, another disease conditioning the psychosocial traits of people [14]. Moreover, in the last years there is an increasing awareness of inflammatory pathologies that are seronegative like spondylarthritis, affecting the cervical area creating a pain situation very similar to TMDs [13].

TMDs are the most common orofacial pain condition of non-dental origin. The etiology of TMDs has been the center of debate over years, therefore many researchers suggest a multifactorial cause. The factors can be stress [14], age [15], gender [16,17], occlusion [18], parafunction [19], airway compromise [20], postural alteration [21], psychological and psychosocial traits [22] and as said before, systemic disease [13].

There is a different prevalence of these disorders in relation to the age group. There is a more frequent onset between the ages of 25 and 40 years (up to 2–4% among those in elder age), and to sex, with a strong female representation (female: male ratio of about 4:1), in particular in the premenopausal age. The reason behind this gender imbalance in the prevalence of TMDs is not clear, but it appears to be influenced by hormonal factors. Studies on animal and human models shown how sex hormones [23] could predispose to TMJ dysfunctions and cartilage wear. High dosages of estrogen progestins have been found in patients diagnosed with TMDs [20,21,24]. However, a unique and definitive link between hormones and TMDs has not yet been identified.

Only a small patients’ percentage who suffer from TMDs pain will seek medical or dental help for the treatment of this condition: when the symptoms are too severe to handle [25]. Cervical spine disorders were shown to be associated with TMDs pain 70% of the time [26].

TMDs can be classified into simplex or complex and acute or chronic as stated by the Diagnostic Criteria for temporomandibular Disorders (DC/TMD) [5].The complexity of these pathologies is noticeable in various aspects: diagnosis, treatment, associated comorbidities and socio-economic impact they entail. In the mid-1970s a new care’s concept was born: the interdisciplinary approach. This integrates a group of specialists jointly dedicated to the study, diagnosis and management of chronic pain conditions [27].

Early detection of TMDs is one of the main concerns to minimize the risk of pain, irreversible articular tissue damage and chronicity, which worsens the prognosis of TMDs. Evidence supports the use of an interdisciplinary approach in patients with TMDs [28] to get better results. Considering a pathology with a multifactorial etiology, the treatment often has to be managed by several specialists: dentist, osteopath, physiotherapist, psychologist, speech therapist, sleep disorder specialist and pain therapist.

Craniomandibular system includes head, neck, and shoulder girdle. In this regard TMJ, muscles, ligaments, fascial connections, together with neural and circulatory innervations are all intimately related. Any disfunction, malocclusion, postural alteration or trauma of the area could possibly lead to a problem at the adjacent structures. The physiological free-way space of the TMJ could be reduced as a consequence of the muscular activity that control the cranio-cervical extension of the head, allowing the elevation and retrusion of the mandible. On the other hand, the mandibular position can be influenced by the tissue elasticity, when the muscular and tendinous connective tissues are stretched as a result of forward head position.

Gnathological treatment integrated with physiotherapy/osteopathy has been shown to be more effective than the dental treatment alone [29] and much better prognosis can be achieved. The physical therapy changes according to the types of patient’s symptoms. Systematic reviews show manual therapy, jaw exercises and postural re-education to be beneficial to decrease TMJ pain, improving mobility and increasing jaw opening, so restoring the function [30,31]. Among the different approaches there are some researches that use a manual approach to treat dysfunctions of the skull, sacrum and totality of the body in order to improve the fluctuation of cerebrospinal liquor, cranial structure, neural function and circulation [32]. Other conservative approaches include low-level laser therapy, electrical stimulation and ultrasounds to reduce inflammation, which further promotes healing of tissues [33]. When pain is the main symptom, it is often necessary to resort to meds therapy. Nonsteroidal anti-inflammatory drugs (NSAIDs) and muscle relaxants are recommended initially, and benzodiazepines or antidepressants may be added for chronic case. It can be helpful to the clinician to be aware of the type of drugs that gave the most relief, as this can give an impulse towards a certain type of diagnosis. If it is a muscle or joint disorder it will have a different response to the various types of drugs. It also allows to distinguish one central pain situation, as there are certain drugs that can condition the Central Nervous System (CNS). In this direction, it is very important for all the therapists involved in the cure of TMDs to have a good knowledge of the meds and their way of acting, even the ones who are not used to prescribe them as the PTs and the OOs [34,35]. Meds alone have not been sufficient to treat the broad spectrum of symptoms related to TMDs, but can represent an aid in a framework of treatment, based on different approaches. The most used molecules are the non-steroidal anti-inflammatory drugs (NSAIDs). In addition, there are also antiepileptics that, as well as antidepressants, are used in chronic forms of pain. These drugs, like pregabalin and gabapentin with off-label use, act on the central component. If they determine a significant improvement of symptomatology, they allow the clinician to quantify the role that the CNS has.

Another category of drugs which, however, is not generally recommended for this type of pain, are the opioids because, acting on the CNS, they determine in addition to the reduction of pain, a marked impairment of the patient’s ability to perform daily activities [36,37].

Osteopathy is a manual therapy, complementary to classical medicine, which treats the patient in a conservative way, without using drugs, by studying the individual as a whole and, rather than aiming to resolve the symptoms, searching for the cause [36]. Positive clinical outcomes were reported for pain reduction, change in autonomic nervous system function, and improvement of sleeping patterns [37]. The use of mandibular myofascial attacks and joint dysfunction’s techniques can reduce pain sensitivity, inflammation and restore oral motor function [38]. In the treatment of myofascial TMDs is also used the acupuncture: a reasonable adjunctive treatment for short-term analgesia in patients with painful TMDs symptom [39].

Given the need for collaboration of different medical figures who treat the same complex pathology, it’s important to investigate on the knowledge and attitudes of two professional figures: osteopaths (OOs) and physiotherapists (PTs). Nowadays it is not clear if there are many PTs and OOs that have enough culture about TMDs and are sufficiently concerned about this health problem.

The aim of this study is to assess confidence levels and training of PTs and OOs treating TMDs and to create awareness regarding the importance of an interdisciplinary approach.

## 2. Materials and Methods

An anonymous survey available both in English and Italian, was shared electronically between therapists from different practices and from different countries. 410 therapists answered the questionnaire, but only 400 answers were considered, because of missing answers and overlap of timing and answers (Supplementary Materials). The questionnaire was composed using Google form (Google LLC., 1600 Amphitheatre Parkway, Mountain View, CA, USA) and the questions were written specifically for this study, and proposed to PTs, OOs and clinicians that have both titles. All collected data were anonymous. Informed consent and acceptance of the privacy policy for the protection of personal data was obtained for all the participants. The questionnaire was diffused through an online form service (Google Form service, Google LLC., 1600 Amphitheater Parkway, Mountain View, CA, USA). No reminders were sent. It was verified that each specialist provided a single answer by checking kind and timing of the responses. All the answers that did not fit those parameters, were excluded from the study. No compensation or benefit was offer to the clinicians in order to complete the survey. Due to the contingency of the COVID-19 pandemic waves, pre-testing was not a viable option. Moreover, limitations of an anonymous questionnaire were taken in consideration, in particular the risk of misunderstanding. Post-Hoc power of the study, considering the difference in probability between the two major groups (PTs 40% and PTs/OOs 58%) of treating occlusal disorders we used the formula for post-hoc dichotomous variables and found a power of 80.4%.

Statisical Analysis: All data were processed using SPSS 25 (IBM Corp. Released 2017. IBM SPSS Statistics for Windows, Version 25.0. Armonk, NY, USA: IBM Corp). Continuous data are expressed as Mean+DS. For dicothomous varibables between three or more groups, it was applied logistic regression, while for continuous data it was applied ANOVA test with Bonferroni Post-hoc.

## 3. Results

**Figure 1:**
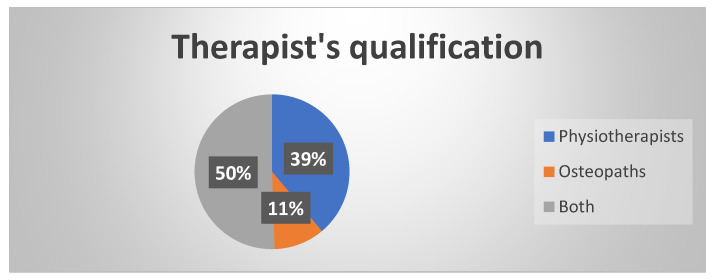
Therapist’s qualification.

Four hundreds answered the questionnaire in an anonymous way. The greatest number of the answers was from Europe (94.5%). One hundred fifty-five were PTs, forty-two were OOs and two hundred had both qualifications (Figure 1). The major problems that they faced in their clinical activity are muscle tightness (61.5%), mouth opening limitation (56%) and disk dislocation (43%) (Table 1). It is present a significant correlation between the profession and the factors investigated (Spearmann e chi2). The clinicians with both degrees (Physiotherapy and Osteopathy) have more probability to investigate the spinal column (*p* < 0.01 R 0.226), the analysis of the masticatory muscles (*p* < 0.05 R 0.162). There are not significant differences in the study of the movements of the mandible, in the auscultation of TMJ noises, in the analysis for the parafunctional habits, in the hearing and studying the clinical history of the patient in a detailed way (only one gave an answer about that).

OOs and clinicians with both degrees showed more interest towards occlusal problems (*p* < 0.01 R 0.170), mandibular/disk dislocation (*p* < 0.01 R 0.239) and muscle contraction (*p* < 0.05 R 0.104) rather than PTs. There were not significant differences considering the treatments of the degenerative disease of TMJ, the limited opening of the oral cavity and the analysis of the trigger points. The therapists with both degrees declared to participate in a continuous way in the education of the patients with TMDs more than the PTs. The latter seemed to spend more time in the TMDs’ patients treatment and education than OOs (*p* < 0.01 R 0.443).

Considering the years of practice and the age, it appeared a linear relationship between the probability of a constant education and years of practice (*p* < 0.01 Variation expected: 1.087/year of practice). The age did not show any statistical variation. Considering the question “how much do you think you know about TMJ and its problems?” there was a statistical significant difference between the OOs (6.41 + 1.55) and the other two groups as the PTs (4.81 + 2.10) and the therapists with both titles (4.02 + 1.78) (ANOVA, Post Hoc di Bonferroni) *p* < 0.01 (general average 4.58 + 2.01). It seemed, according to the question “Do you feel confident treating a patient with TMDs?”, that clinicians with both status were more confident to treat TMDs than the OOs, who were more convinced than PTs (Table 1).

## 4. Discussion

TMDs are a syndrome that according to the recent literature should be assessed through the diagnostic criteria. These ones differently from the occlusal theory, consider the multifactorial etiology of the disorder. In this study, it is evident that other therapists, like PTs and OOs started already to treat and improve quality life of patients with TMDs. Patients with TMDs usually have a very low quality life. It is already highlighted that manual therapy can improve the signs and symptoms [40]. According to the results of this manuscript, the majority of the therapists are used to treat muscle tightness: there are studies that highlight how manipulation, massage and myofascial release produce a higher relief than placebo already [29,30]. This aspect is very important, because it can reduce the use of muscle relaxants and similar meds. Furthermore, it is an important aid when the meds start to have a lower benefit, because of the prolonged and excessive use [35]. The manual therapy appears to be effective on the masticatory muscles, as well as on the upper cervical spine, which is an area where TMDs patients refer pain [41]. The questionnaire evidences that all the kind of therapists treat the mouth opening limitation which, is due to the muscle contraction in most cases. Both the clinicians, to treat this condition, need to have a specific knowledge of the stomatognathic system and the facial anatomy. The most recent techniques to release the tightness are various. Among the osteopathic procedures, the Muscle Energy Technique (MET) involves restrictive barriers without putting excessive effort on the body. Guided by the operator, the patient is asked to perform a muscular effort in a specific direction, opposing a counterforce. The TMJ can be manipulated through the Myofascial Release (MFR). In this procedure the operator palpates the joint and the soft tissue and examine the presence of tightness and looseness while twisting and compressing the structures. Balanced Ligamentous Tension (BLT) is instead a technique which focus on the ligaments guide of the TMJ movements, and it is developed exaggerating the TMJ disfunction in both a passive and active way [42]. There are articles that confirm that manual therapy sometimes associated with oral splint, mainly used at night, can improve and resolve disk dislocation [43].The kinds of splint used in these situations are different, they can be plate ones, similar to the Michigan bite or the stabilization plaque or others, like the anterior splint [44,45]. Sometimes the reduced range of motion and the muscle weakness are associated with active myofascial trigger points. The quantity of the maximal mouth opening and concomitant acute effects on masticatory muscles’ activity have been shown to be treated by different manual approaches, like compression techniques. This seems to be effective in myofascial trigger points recovery [46].

From the results of this study, it seems that OOs and clinicians with both degrees appear to be careful to TMDs’ signs and symptoms, they look at the occlusal state and muscle balance. Differently from the PTs they consider all the other biological structures linked to the TMJ, like the spine: it is likely that they are more used to consider all the whole human body. This study highlights OOs appear to have a major knowledge about TMDs, probably due to the different approach to body’s pathologies and anatomy. Moreover, the clinicians with both degrees have the greatest self-confidence to treat TMDs. This can be explained by the fact that the techniques and the knowledge of PTs and OOs can be complementary and give more therapeutic instruments. Furthermore, as it is affirmed strongly in the literature, TMDs need an interdisciplinary approach. In this study there’s an interesting aspect: both the therapists demand the patients to other specialists, in particular orthodontists are the ones much looked for. Orthodontists have a higher knowledge of the occlusal principles and can understand if there is any imbalance. There are studies who claimed that some cases of TMDs when treated with occlusal therapy and a manual approach can improve in significant way [47]. The techniques adopted by the therapists are vary, in this study they consider the manual one, which appear the one most used for this health problem (75.5%). The second one more used is the postural treatment (48.5%) and the third is the proprioceptive one (43%). Those have an evidence in literature, in particular the posture therapy is considered a good way to reduce the signs and symptoms of TMDs, because there are case where pain and disfunction of the upper area of the spine is caused by the posture of the rest of the body [48]. The proprioceptive therapy sometimes produces good results, in term of disrupt the muscle pattern and can determine a relief which, in most patients, does not last for a long period [49]. There are new treatments that can be adopted to reduce the impact of TMDs, but these should be analyzed and assessed to determine where they can be useful.

Tongue posture can cause an alteration of the function of muscles that are tied to the hyoid bone, so a figure that recently start to be involved in orofacial pain is the speech therapist. The myofunctional therapy can be adopted when there is a dysfunction of the facial muscles and the tongue’s ones. This approach can be used even by dentist, orthodontists, PTs, and OOs too [50,51].

According to what has already been said, it is important to make a correct diagnosis and refer the patient to the required clinical specialist.

A teamwork is very important to have an interdisciplinary approach. The co-work is extremely important for better outcomes, avoiding the misdiagnosing, and lowering the cost. Often due to the absence of a team, the patient risks to undergo treatments that give him a temporary benefit but are not resolutive. The lack of this bring to a non-lasting solution.

Limitations of the study: The study has limitations caused by the complexity of the pathology. All the limits of an anonymous questionnaire were considered. The questionnaire was compiled specifically for this study and, because of the contingency of the COVID-19 pandemic waves, pre-testing was not a viable option. A problem of the study is the possibility of misunderstanding the questions and, consequently, giving wrong answers. Another possible limit is the origin of the sample. This is characterized by European origin mainly, while it is quite known that the figure of physiotherapist has other features in other continents, in particular in the U.S. The sample of OOs is quite less than the one of PTs and this can be another problem. According to the Checklist for Reporting Results of Internet E-Surveys (CHERRIES), the result of a web survey using an anonymous questionnaire cannot be evaluated like a certain result, but it can only be considered a hypothesis that should be validated in a more checked study. However, this study cannot be considered a pure web survey, because it was widespread through clinical practices.

## 5. Conclusions

The importance of the interdisciplinary work between dentistry, in particular orthodontists, and PTs/OOs for TMDs treatments is quite evident, considering the results obtained. It is essential to have a shared treatment plan where the physical therapy helps in pain relief and dentistry treats the disorders related to the stomatognathic system. From this study it seems that PTs and OOs are already used to seek more orthodontic consulting for their patients than the opinions of other kinds of clinicians. The hypothesis inferred from the study is that it is growing the importance given to TMDs by PTs and OOs. It is reasonable to affirm that more possibilities should be offered by dentists and orthodontists to OOs and PTs to get in touch with dysfunctional patients, to improve their quality of life. Eventually this study highlights OOs and all the clinicians with this qualification, have a higher confidence in treating patients with TMD than the others. These findings can be important for academics, educators and healthcare professionals to improve TMDs education, awareness and knowledge.

## Figures and Tables

**Figure 2:**
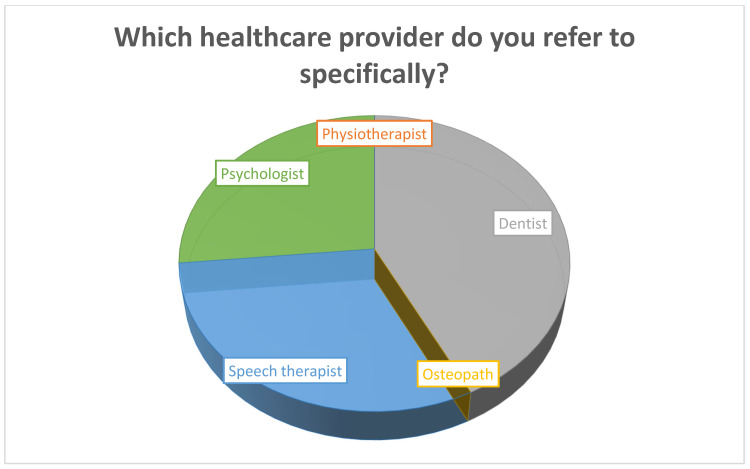
Which healthcare provider/speciality do you refer to specifically?

**Table 1:** Demographic details and results.

	Frequency	Proportion	
Assessment—[From the options below, what do you normally include in the evaluation of your patients with TMD?]			
Jaw movements	279	69.8	
TMJ palpation	269	67.3	
Masticatory muscles	262	65.5	
TMJ sound	234	58.5	
Cervical spine	209	52.3	
Occlusion	196	49.0	
Parafuncion	167	41.8	
History	1	0.3	
Problem—[What type of TMD have you evaluated and/or treated? (select all that applies)]			
Muscle tightness	246	61.5	
Mouth opening limitation	224	56.0	
Dislocation	174	43.5	
Trigger points	155	38.8	
TMJ Degeneration	135	33.8	
Malocclusion	128	32.0	
Profession—[What is your profession?]			
Physiotherapist	155	38.8	
Osteopath	42	10.5	
Both	200	50	
DId not respond	3	0.8	
Geograpical area—[Where do you live?]			
Africa	2	0.5	
North America	9	2.3	
Central America	2	0.5	
South America	2	0.5	
Europe	378	94.5	
Asia and Middle east	3	0.5	
Oceania	1	0.3	
Did not respond	3	0.8	
	Mean ± SD	Range	
Age	39.9 ± 10.8	21–69	
years of Practice [How many years have you been practicing?]	14.9 ± 10.2	0–47	
Gender			
Male	214	53.8	
Female	184	46.0	
Did not respond	2	0.5	
[Which healthcare provider/speciality do you refer to specifically?] Figure 2			
Orthodontist	213	53.3	
Physiotherapist	159	39.8	
Dentist	154	38.5	
Ostheopath	121	30.3	
Speech therapist	112	28.0	
Psychologist	96	24.0	
Oral surgeon	89	22.3	
[How much do you think you know about TMJ and its problems? (1–10)]	Physiotherapists	Osteopaths	Both
	4.81 ± 2.10	6.41 ± 1.55	4.02 ± 1.78
[Do you feel confident treating a patient with TMD? (1–10)]			
*p* < 0.05	96/155 (61.9%)	30/42 (71.4%)	185/200 (92.5%)

## Data Availability

The data that support the findings of this study are available from the first author (S.S.) and corresponding author (M.T.P.) upon reasonable request.

## Supplementary Materials

The following supporting information can be downloaded at: https://www.mdpi.com/article/10.3390/dj10110210/s1.
