# Supplementary material for: Physiotherapists and Osteopaths’ Attitudes: Training in Management of Temporomandibular Disorders
Source: Dent J (Basel). 2022 Nov 4;10(11):210. doi: 10.3390/dj10110210 (PMC9689146; doi:10.3390/dj10110210)
Supplement: Supplementary file 1 [file dentistry-10-00210-s001.zip › s1.pdf]

- Età (inserire anni) Age (enter in years)

18 : 0.2 %
20: 0.5 %
22: 1.2%
23: 1.5%
24 : 2.2 %
25: 3.2 %
26:2.4 %
27: 3.4 %
28: 2.2 %
29: 2.9 %
30:3.4%
31: 0.2%
32: 2.4 %
33: 2.2%
34: 4.2%
35: 2.4%
36: 4.6%
37: 2.7 %
38: 1.1%
39: 2.7%
40: 2.7%
41: 3.4%
42: 2.2 %
43: 2.7%
44: 3.4%
45:3.2 %
46: 2.9%
47: 3.9%

48: 2.2%
49: 1.5%
50: 1.5%
51: 1.2%
52: 1.5%
53: 1.7%
54: 0.5%
55 :2.2%
56: 2 %
57: 1.2%
58: 0.5%
59: 1.5%
60: 1%
61: 0.12%
62: 0.2%
63: 0.2%
64: 0.5%
65: 0.5%
66: 1%
69: 0.2%

Nei pazienti con disordini temporomandibolari,quali di questi aspetti valuti? (puoi scegliere più di una risposta)
From the options below,what do you include the evalutation of your patients with TMD?

Colonna cervicale – Cervical Spine : 53.9 %
Palpazione dell’ATM- TMJ palpation : 68.5 %

Palpazione dei muscoli masticatori- Palpation of masticatory muscle 67 %
Movimenti mandibolari in apertura e chiusura- Jaw movements during opening / closing 70.9 %
Rumori articolari – TMJ sounds 59.6 %
Segni di parafunzione - Signs of parafunctional habit: 42.4 %
Occlusione dentale – Dental occlusion: 49.3 %
Psico-emotività, storia clinica – Psycho – emotionality, clinical history: 0.2 %
Sempre una valutazione generale- Always a general assessment: 0.2 %
Valutazione della funzione della lingua e deglutizione - assessment of tongue fuction and swallowing : 0.2 %
Osso ioide,lingua – hyois bone,tongue 0.2 %
Cranio- Skull: 0.2 %
Catene miofasciale – Myofascial chains: 0.2 %
Tutte quelle precedenti – All of the above: 0.2 %
Disfunzione del cranio- Skull dysfunction : 0.2 %
Rumori articolari, dolore alla masticazione :Joint noises , pain on chewing : 0.2%
Aspetto lingua e muscolatura – appearance of tongue and mucles: 0.2 %
Piede - foot : 0.2 %
Dorsale,mediastino- dorsal mediastinum : 0.2%
Stato della percezione dolore- state of pain perception: 0.2%
Ossa craniche e mtr: Cranial bones and mtr 0.2%
Postura- Posture: 0.2%
Diaframma cranica,toracico – Cranial diaphragm thoracic: 0.2 %
Rachide in toto,postura : Spine as a whole,posture : 0.2 %

Assetto posturale globale – Overall postural attitude: 0.2 %
Punti miofasciali – Myofascial points: 0.2 %

-Quale tipo di disordini temporo mandibolari hai valutato maggiormente? (Puoi scegliere piu di una risposta) – What type of TMD have you evalutated and / or treated ? (Select all that apply)

Dislocazione del disco dell’ATM – TMJ disc dislocation of the: 43.9 %
Degenerazione del disco: Disc degeneration 34 %
Ipermobilità dell’ATM- Hypermobility of TMJ 32.3 %
Limitazioni dell’apertura della bocca – Limitations on opening the mouth : 57.8 %
Contrattura muscolare- Muscle contracture : 63.5 %
Punti grilletto- Trigger points: 0%
Alterazione occlusale- Occlusal alteration: 32.5 %
Punti Grilletto dei muscoli- Muscle trigger point: 39.7 %

Che tipo di disordini temporomandibolari presentavano I pazienti che hai valutato? (Puoi scegliere piu di una risposta) – What stage of tmd have your patients withduring evaluation? (select all that apply)
405 risposte

Acuto- Acute: 44.4 %
Subacuto- Subacute: 42%
Cronico - Chronic: 64.9 %

Con quali tecniche tratti I pazienti affetti da disordini temporo mandibolari ? (Puoi scegliere piu di una risposta) When treating patients with TMD, what interventions do you use? (Select all that apply)
401 risposte

Laser- Laser: 15.5 %
Terapia Manuale- Manual therapy: 75.6 %
TENS: 23.2 %
Terapia Propriocettiva Proprioceptive therapy : 43.1 %
Terapia postural- Postural therapy: 48.6 %

A Quale tipo di specialista sei solito riferire i tuoi pazienti ? (puoi scegliere piu di una opzione)
Which healthcare provider / Specialty do you refer to specifically ? (Select all that apply)

Dentista generalista- General dentist: 39.6 %
Ortodontista – Orthodontis : 54 %
Fisioterapista specializzato- Specialized Physiotherapist: 40.8 %
Osteopata- Osteopath 30.3 %
Medico generico- General practitioner 22.9 %

Chirurgo Orale- Oral surgeon: 22.4 %
Logopedista- Speech therapist : 28.6 %
Psicologo- Psychologist : 24.1 %
Altre opzioni- Other Options: 0%

Quanto pensi di conoscere l'ATM e le sue problematiche ?
How much do you think you know about TMD and its problems ?
Da 1 a 10.

1: 2%
2: 13.3 %
3: 21.1 %
4: 17.7 %
5: 13%
6: 13.8 %
7: 10.1%
8: 6.1%
9: 1.7 %
10: 1.2 %

Da quanti anni pratichi?- How many years have you been practing ? 10
20
5
22
1
3
2
30
13
4
7
15
12
6
14

16
11
26
17
19
9
25
27
23
21
18
0
8
24
40
31
5

1
35
2
30
33
28
15
10
29
2
36
38
25
1
3
5
8
11
10
2
1
12
10
30
18
0.7
9
22
23
7
8
4
8
27
7
15
5
21
34
37
41
42
43
47
12
14
