# Supplementary material for: Physiotherapists and Osteopaths’ Attitudes: Training in Management of Temporomandibular Disorders
Source: Dent J (Basel). 2022 Nov 4;10(11):210. doi: 10.3390/dj10110210 (PMC9689146; doi:10.3390/dj10110210)
Supplement: Supplementary file 1 [file dentistry-10-00210-s001.zip › s2.pdf]

Sesso-Gender 408 risposte

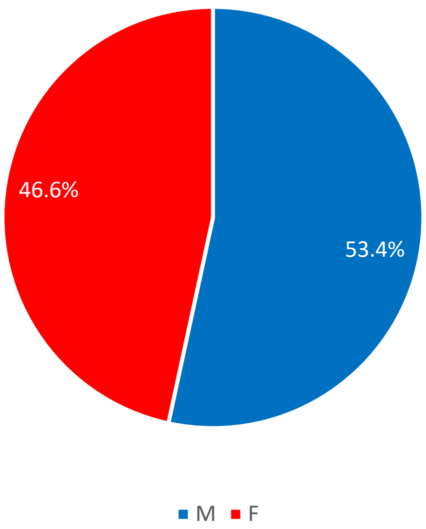

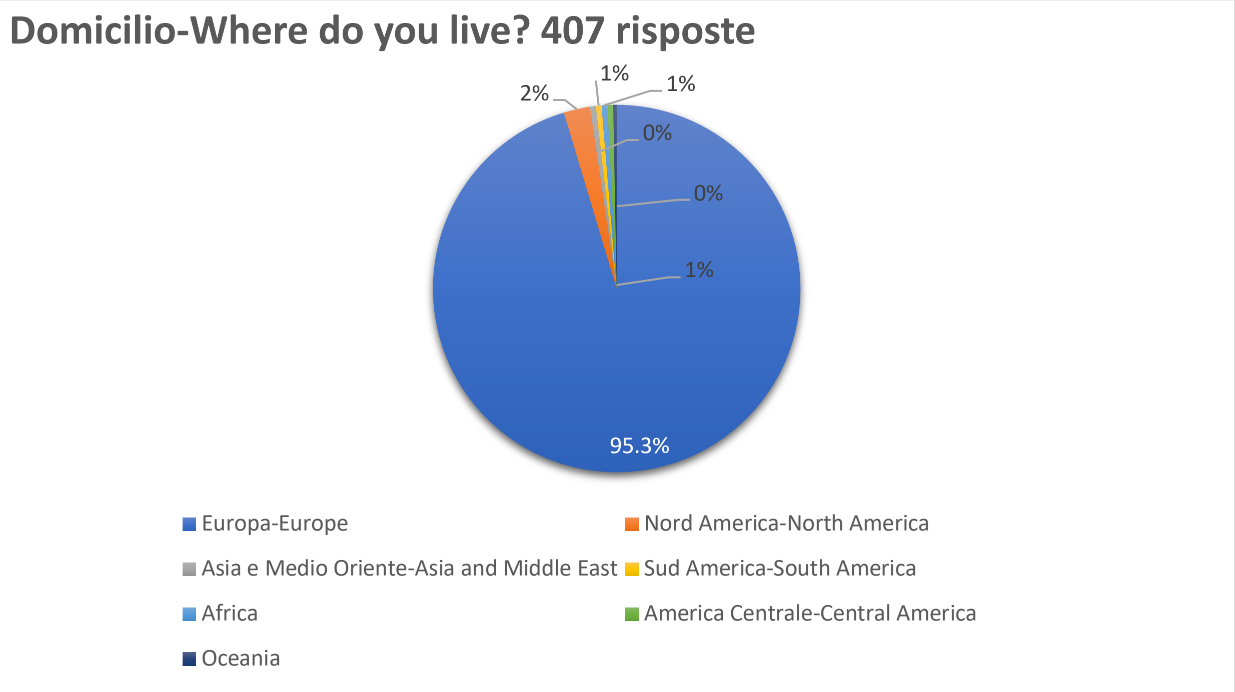

Hai conseguito la laurea in fisioterapia?--Did you have bachelor’s degree in Physical Therapy?
408 risposte

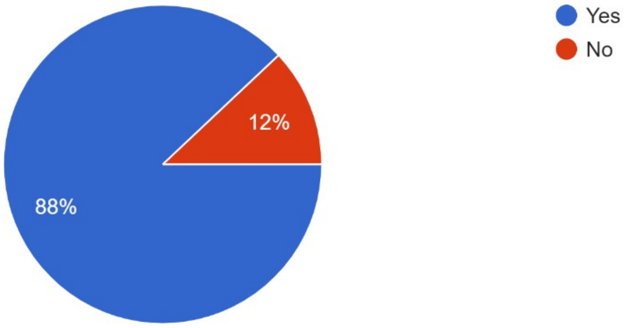

Hai conseguito la laurea in osteopatia?--Did you have bachelor’s degree in ostheopaty?
407 risposte

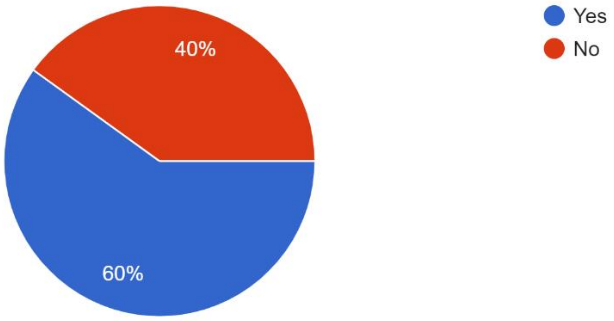

Qual è la tua qualifica?-What is your profession? 407 risposte

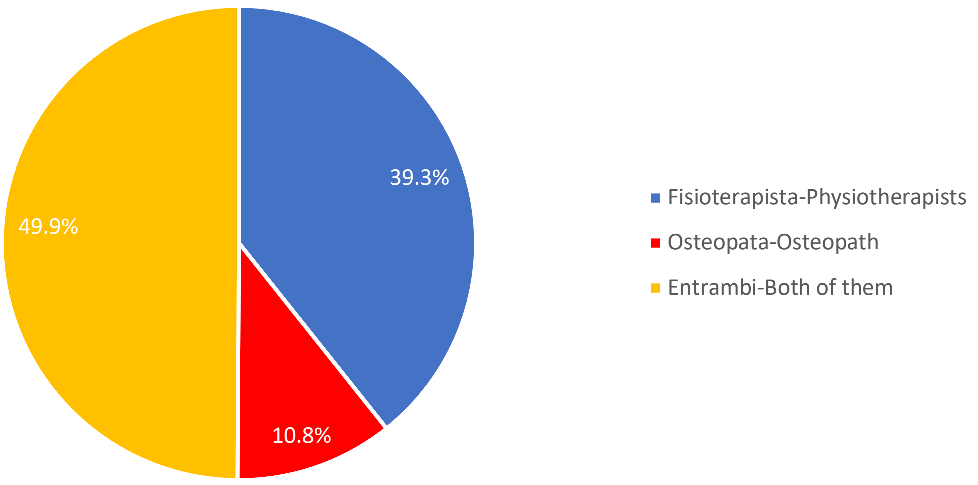

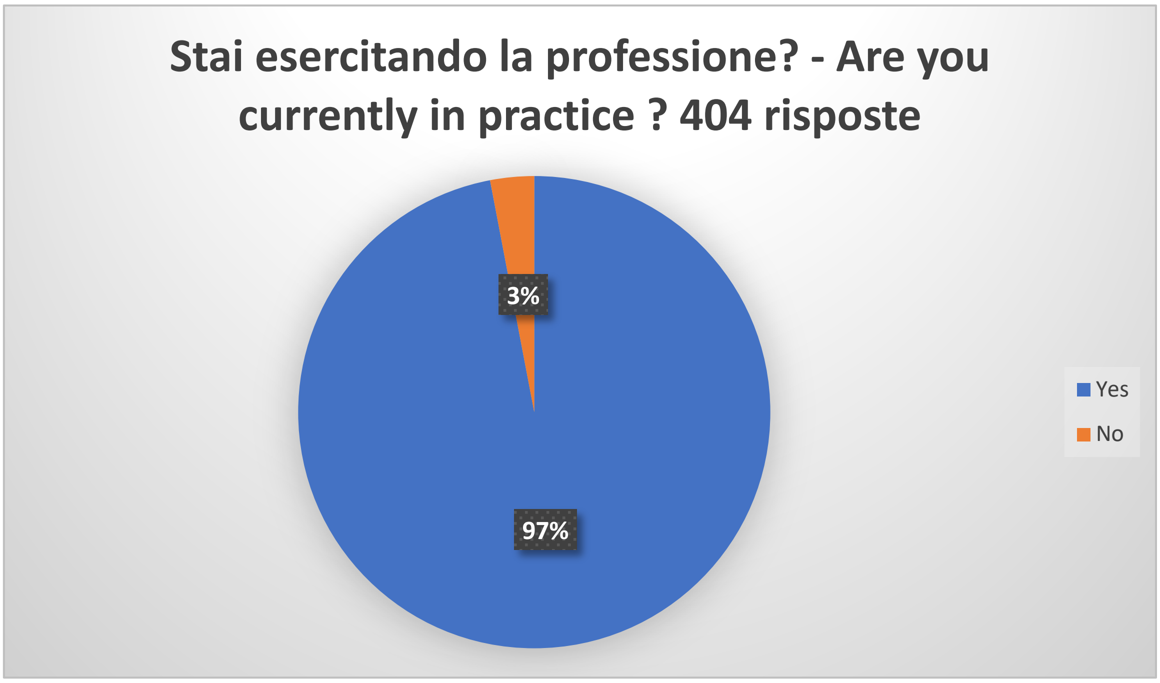

Hai mai seguito un corso di formazione continuo su disturbi temporo-mandibolari (TMD)?--Have you ever taken a continuing education course on temporomandibular disorders (TMD) ?
406 risposte

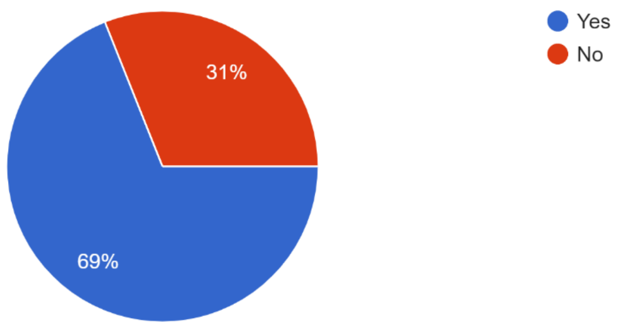

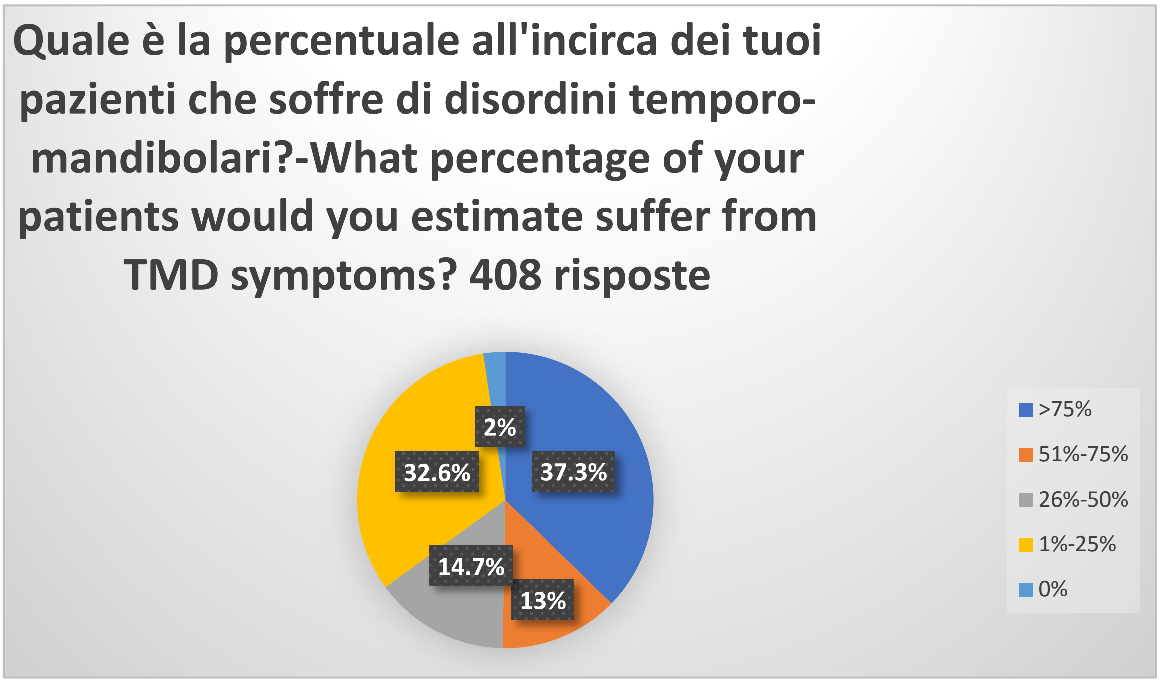

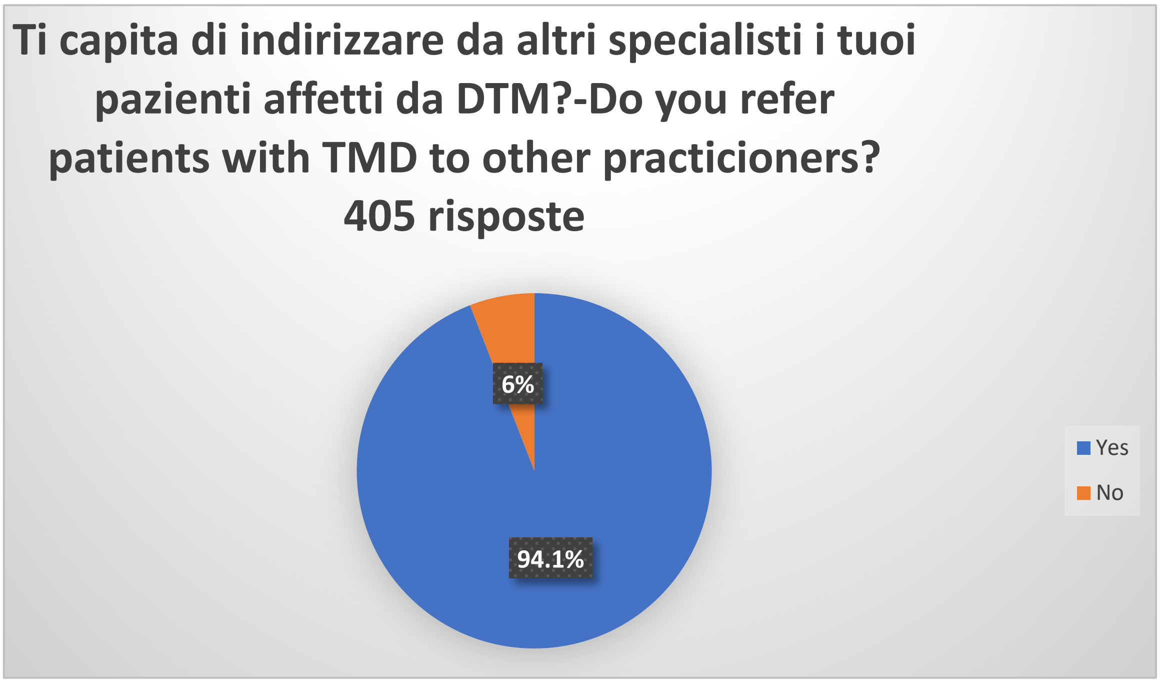

Quale percentuale di pazienti riferisci ad altri specialisti per altre tipologie di trattamento?-What percentage of these patients do you refer to other specialists for other kinds of therapies? 404 risposte

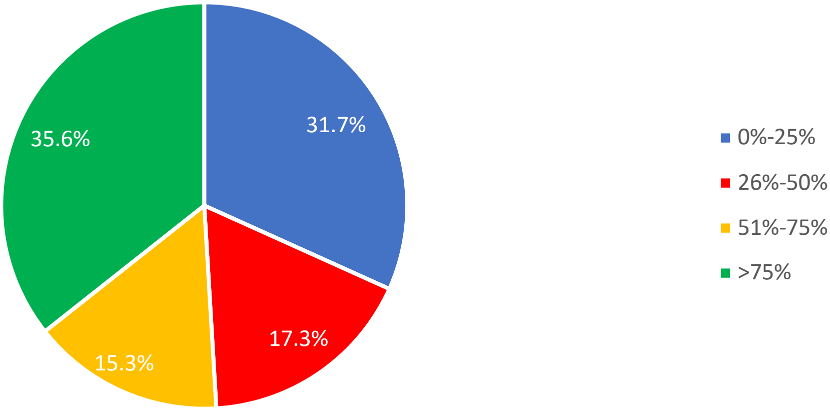

Before this survey,were you aware that physiotherapists/ osteopath can treat patients with TMD,for exameple by reducating movement and restoring masticatory function?

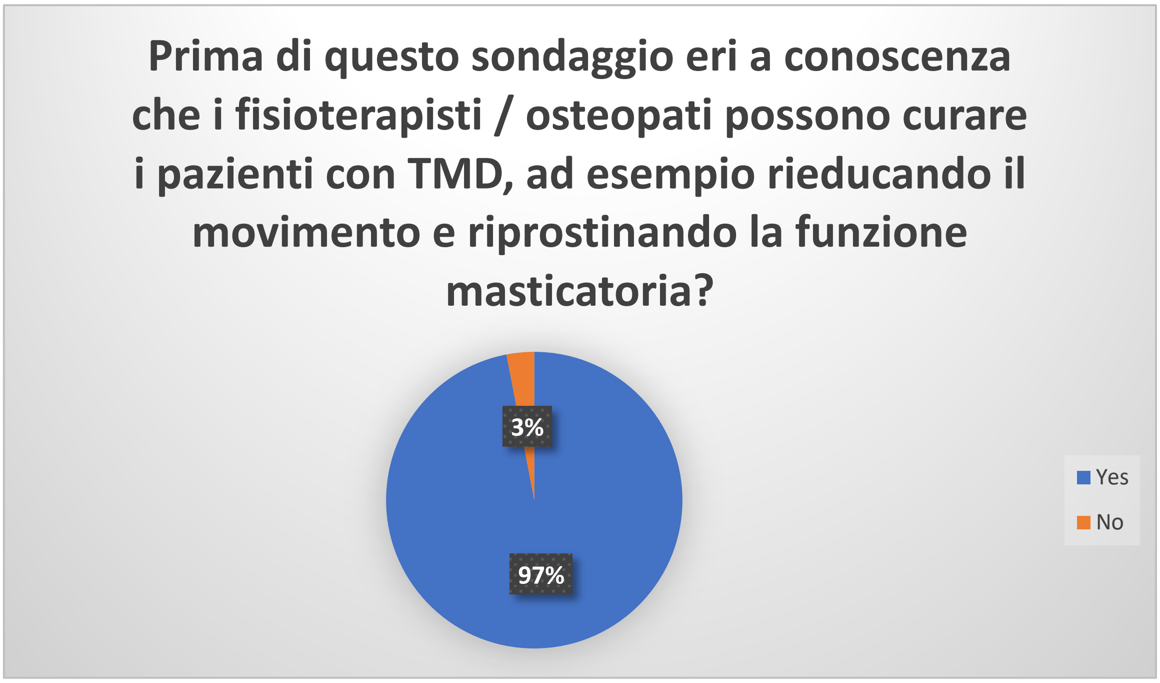

Ritieni di aver ricevuto informazioni adeguate/educazione sulla TMD durante il percorso universitario?-Do you feel that you received adeq… information/education on TMD while university? 407 risposte

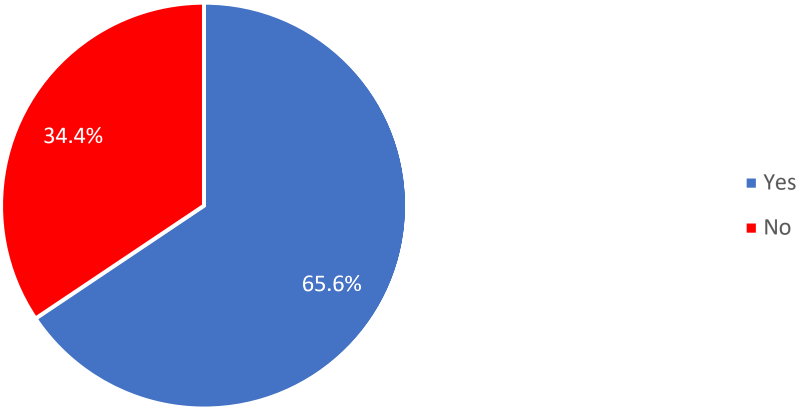

Ti senti sicuro nel trattare un paziente con DTM?-Do you feel confident treating a patient with TMD?406 risposte

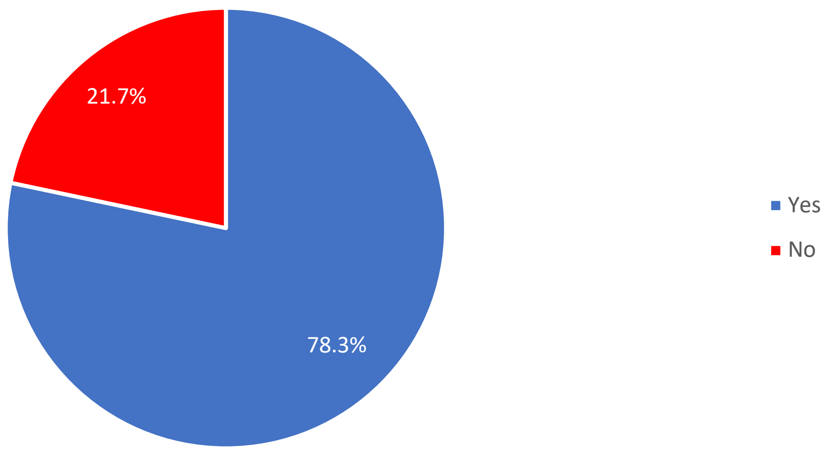

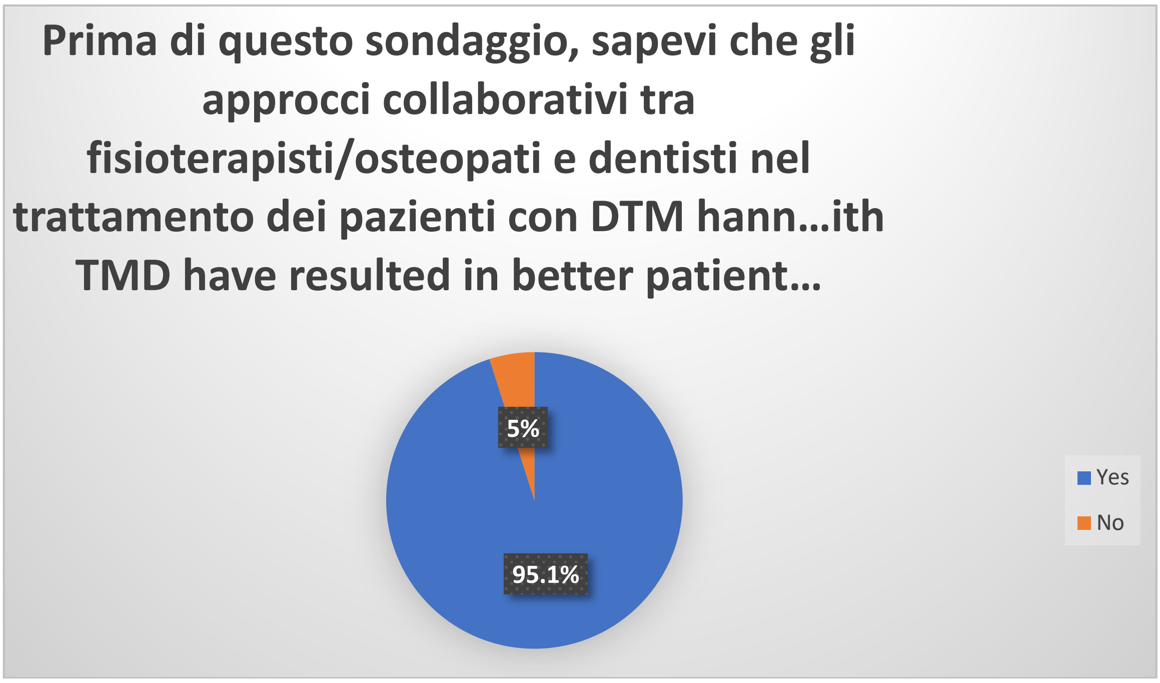

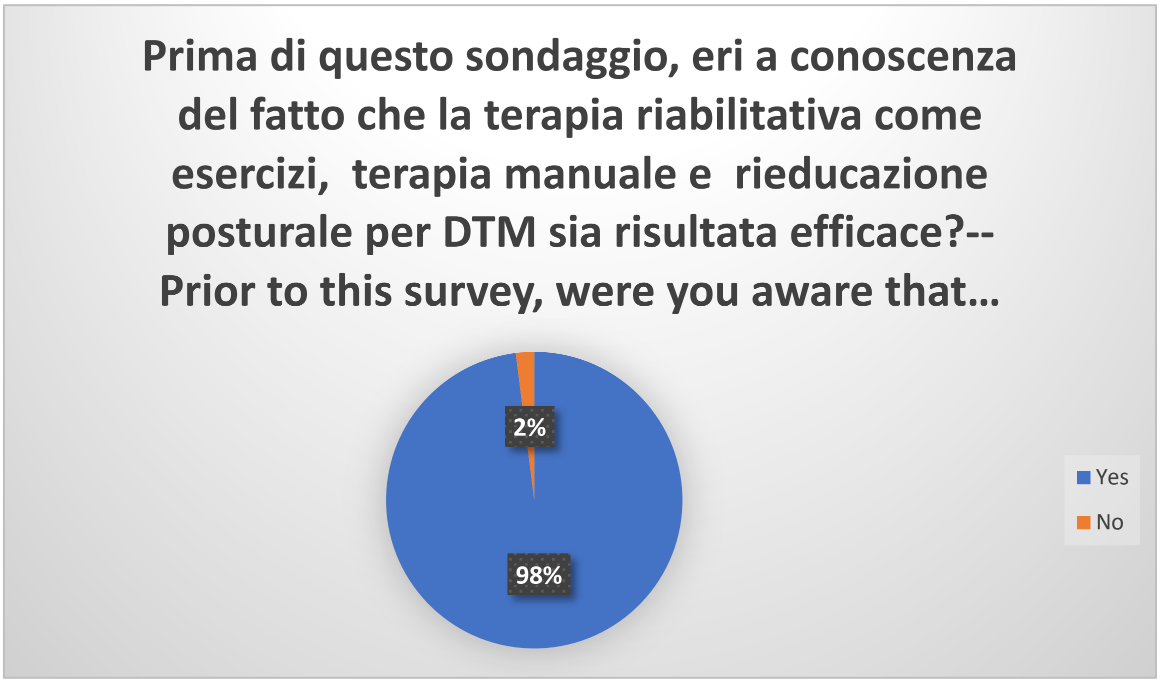
Prima di questo sondaggio, eri a conoscenza del fatto che la terapia riabilitativa come esercizi, terapia manuale e rieducazione posturale per DTM sia risultata efficace?--Prior to this survey, were you aware that...
2%
98%
Yes
No

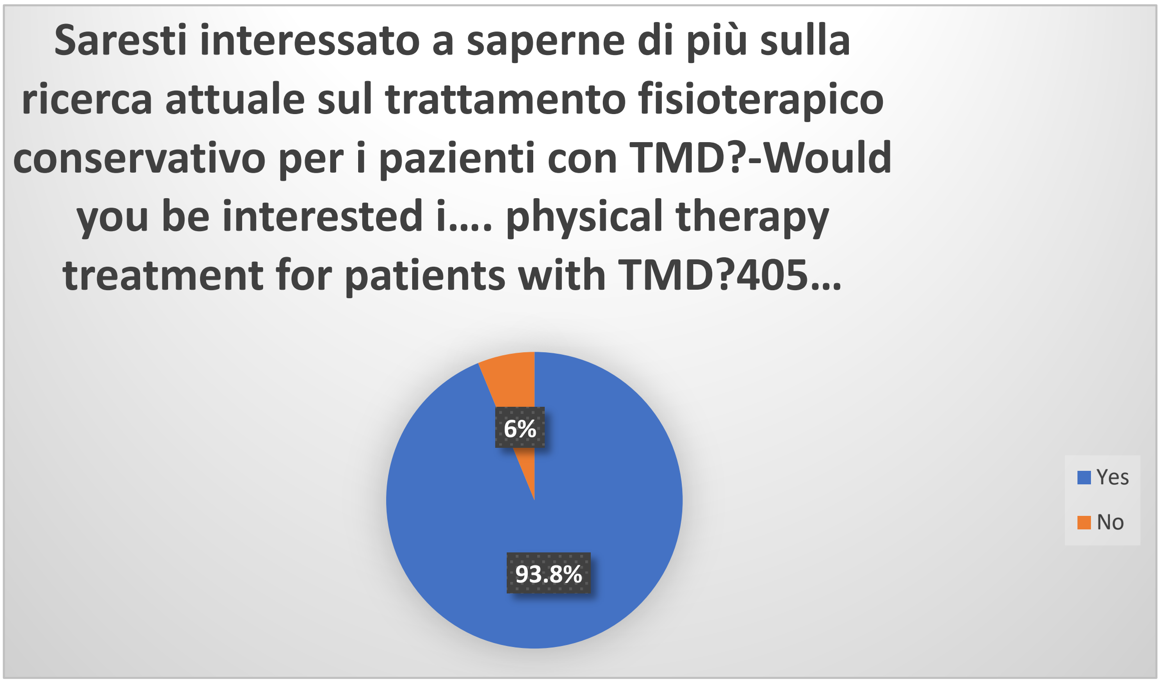
Saresti interessato a saperne di più sulla ricerca attuale sul trattamento fisioterapico conservativo per i pazienti con TMD?-Would you be interested i.... physical therapy treatment for patients with TMD?405...
6%
93.8%
Yes
No
